# Assessment of indicators of vitamin A status in non-cirrhotic chronic hepatitis C patients

**DOI:** 10.1590/1414-431X20154785

**Published:** 2015-11-17

**Authors:** R.C. Santana, A.A. Machado, A.L.C. Martinelli, A.A. Jordão, L.N.Z. Ramalho, H. Vannucchi

**Affiliations:** 1 Divisão de Infectologia, Departamento de Clínica Médica, Hospital das Clínicas, Faculdade de Medicina de Ribeirão Preto, Universidade de São Paulo, Ribeirão Preto, SP, Brasil; 2 Divisão de Gastroenterologia, Departamento de Clínica Médica, Hospital das Clínicas, Faculdade de Medicina de Ribeirão Preto, Universidade de São Paulo, Ribeirão Preto, SP, Brasil; 3 Divisão de Nutrologia e Nutrição, Departamento de Clínica Médica, Hospital das Clínicas, Faculdade de Medicina de Ribeirão Preto, Universidade de São Paulo, Ribeirão Preto, SP, Brasil; 4 Departamento de Patologia, Hospital das Clínicas, Faculdade de Medicina de Ribeirão Preto, Universidade de São Paulo, Ribeirão Preto, SP, Brasil

**Keywords:** Vitamin A, Hepatitis C, Retinol, Retinol binding protein, Liver fibrosis

## Abstract

Subjects with chronic liver disease are susceptible to hypovitaminosis A due to several factors. Therefore, identifying patients with vitamin deficiency and a requirement for vitamin supplementation is important. Most studies assessing vitamin A in the context of hepatic disorders are conducted using cirrhotic patients. A cross-sectional study was conducted in 43 non-cirrhotic patients with chronic hepatitis C to evaluate markers of vitamin A status represented by serum retinol, liver retinol, and serum retinol-binding protein levels. We also performed the relative dose-response test, which provides an indirect estimate of hepatic vitamin A reserves. These vitamin A indicators were assessed according to the stage of liver fibrosis using the METAVIR score and the body mass index. The sample study was predominantly composed of male subjects (63%) with mild liver fibrosis (F1). The relative dose-response test was <20% in all subjects, indicating vitamin A sufficiency. Overweight or obese patients had higher serum retinol levels than those with a normal body mass index (2.6 and 1.9 µmol/L, respectively; P<0.01). Subjects with moderate liver fibrosis (F2) showed lower levels of serum retinol (1.9 *vs* 2.5 µmol/L, P*=*0.01) and retinol-binding protein levels compared with those with mild fibrosis (F1) (46.3 *vs* 67.7 µg/mL, P<0.01). These results suggested an effect of being overweight on serum retinol levels. Furthermore, more advanced stages of liver fibrosis were related to a decrease in serum vitamin A levels.

## Introduction

The liver plays a major role in vitamin A (retinol) metabolism. This organ is involved in serum retinol uptake after intestinal absorption from nutritional sources, as well as its storage and continuous supply to target tissues. Hepatic stellate cells (HSCs) and hepatocytes are the main cell types involved in this process. HSCs are responsible for the storage of 70% to 90% of the total liver vitamin A. Hepatocytes are mainly involved in the uptake of retinyl esters from chylomicrons, transfer of retinol to HSCs, and synthesis of retinol-binding protein (RBP) (1). In chronic liver diseases, HSCs become activated, losing their retinoid content, and produce an extracellular matrix, which is responsible for liver fibrosis (2). Other factors, such as decreased vitamin intake or absorption, and excessive alcohol consumption, may place cirrhotic patients at risk for hypovitaminosis A, which could benefit from vitamin supplementation. Conversely, when hepatic retinol storage is not depleted, prolonged vitamin A supplementation in subjects with liver disease may induce hepatotoxicity (3). Therefore, estimating vitamin A status is important to distinguish between subjects that have retinol deficiency and those for whom vitamin supplementation is not recommended.

Estimating body vitamin A stores with commonly available methods is a challenging task, which should be performed considering the advantages and limitations of the available laboratory tests. Determination of serum retinol levels is extensively used as a noninvasive and relatively simple test. However, blood retinol levels are under strict homeostatic control and only reliably reflect hypovitaminosis A at the extremes of deficiency. Because plasma retinol is transported coupled to RBP at a 1:1 ratio, serum RBP quantification reflects serum retinol concentrations. Direct quantitation of hepatic retinol best reflects the body's vitamin A reserves. However, the indications of this method are extremely limited and depend on obtaining hepatic tissue by biopsy. Therefore, this procedure is not indicated, except for concomitant diagnostic evaluation of hepatic disease. The relative dose-response (RDR) test has been proposed as a less invasive method, which quantifies relative increases in serum retinol concentrations by comparing the basal fasting level with another sample taken after an oral dose of retinol palmitate. The magnitude of the increase in plasma retinol concentrations provides an indirect estimate of hepatic vitamin A reserves (4).

Studies concerning retinol metabolism when there is chronic hepatitis C (CHC) present have gained increasing relevance. Vitamin A exerts antioxidant protection against oxidative stress, which plays an important role in the pathophysiology of liver disease induced by hepatitis C virus (HCV) infection (5). Furthermore, vitamin A deficiency has been associated with non-response to interferon-based antiviral therapy (6). The majority of studies that evaluated vitamin A status in liver diseases have been conducted in individuals with cirrhosis of various etiologies that were evaluated concomitantly. Relatively few studies have been performed in CHC patients, especially in non-cirrhotic patients. Therefore, the present investigation aimed to evaluate vitamin A status in non-cirrhotic CHC patients according to serum retinol and RBP concentrations, liver retinol concentrations, and the RDR test.

## Material and Methods

### Study design and population

A cross-sectional study was conducted on adult CHC patients who were monitored at the Hospital das Clínicas, Faculdade de Medicina de Ribeirão Preto, Universidade de São Paulo, Brazil. The patients were invited to participate in the study during pretreatment evaluation, from September 2006 to September 2007. A total of 43 subjects were included in the analysis.

Diagnosis of CHC was based on the positive detection of anti-HCV antibodies and was confirmed by HCV-RNA detection in peripheral blood by qualitative polymerase chain reaction (Amplicor¯ qualitative test; Roche Diagnostics GmbH, Germany). Liver biopsy was indicated by the attending physician according to pretreatment evaluation protocols and independently of the study.

Patients who had taken vitamin A supplements during the last 3 months, who were co-infected with HIV and/or with the hepatitis B virus, or who had acute inflammatory or infectious diseases were excluded from the study. We also excluded patients with clinical, laboratory, or histological evidence of cirrhosis or severe fibrosis by liver biopsy analysis.

Patients were weighed and measured using a high-precision scale. Body mass index (BMI, kg/m^2^) was calculated. Laboratory tests were performed over the month prior to the liver biopsy. These results, including alanine aminotransferase levels, serum albumin levels, and the international normalized ratio, were obtained from the patient's medical records. A mean daily alcohol consumption record was also obtained from each patient. The Research Ethics Committee of the institution approved the study protocol. All subjects gave written informed consent to participate.

### Assessment of liver fibrosis

An ultrasound-guided percutaneous liver biopsy was performed in all of the patients. A fragment of the obtained liver tissue sample was used for histopathological analysis. The hepatic stage of fibrosis was determined by the METAVIR score (7), which classifies fibrosis into five categories: F0 (absence of fibrosis), F1 (mild fibrosis), F2 (moderate fibrosis), F3 (severe fibrosis), and F4 (cirrhosis).

### Analysis of serum retinol and RBP concentrations

On the day of the liver biopsy, 10 mL of venous blood was collected for fasting serum retinol and RBP quantitation. Blood collection was performed using tubes that were protected from light exposure. Serum was immediately separated after centrifugation (3000 *g*, 10 min) and stored at -20°C prior to analysis. For retinol quantification, a serum sample (500 µL) was mixed with 1 mL of ethanol and 1 mL of n-hexane was added after mechanical agitation. After centrifugation (3000 *g*, 10 min), 500 µL of supernatant was evaporated and reconstituted with the mobile phase (methanol 10%, dichloromethane 20%, acetonitrile 70%). Serum retinol was analyzed at 325 nm using high-performance liquid chromatography. A Shimadzu 6AV spectrophotometric detector (Japan) equipped with a 25 × 0.46-cm inner diameter Shim-pack CLC-ODS column (Japan) was used. The high-performance liquid chromatography reagents were from Merck (Germany). Serum retinol results are reported as µmol/L.

Serum RBP levels were determined by ELISA using the E-80RBP kit (Immunology Consultants Laboratory, USA). The results are reported as µg/mL.

### RDR test

In addition to quantification of fasting serum retinol (R0), another fasting venous blood sample was collected 5 h after administration of 675 µg of oral retinol palmitate (Arovit¯, Roche) for determination of serum retinol levels (R_5_). As previously described, blood was collected at ambient temperature and was protected from ultraviolet light exposure. Serum was immediately separated by centrifugation (3000 *g*, 10 min) and stored at -20°C until analysis.

The RDR test was calculated using the following formula (8): RDR (%) = (R_5_ - R_0_) × 100 / R_5_. RDR test results >20% were considered to be positive, indicating vitamin A deficiency (4).

### Determination of liver retinol levels

Free hepatic retinol was quantified in the needle biopsy samples. The liver tissue fragments were protected from light and immediately frozen at -70°C. During analysis, these fragments were homogenized (Turrax MA-102/MINI, Marconi, Brazil) in 1 mL of ethanol. After agitation, 2 mL of n-hexane was added and the samples centrifuged (3000 *g*, 10 min). Supernatants were evaporated and reconstituted with the mobile phase. Finally, 100 µL of mixture was injected into the chromatographic column. The results are reported as µg/g liver tissue and represent free retinol, rather than total hepatic retinol (total hepatic retinol = free retinol + esterified retinol).

### Statistical analysis

Statistical analysis was performed using the SAS software for Windows (Release 9.2. SAS Institute, USA, 2011). Graphs were created using the R software version 3.1.1. Descriptive analysis was performed to report continuous variables as medians and ranges or means±SD, and categorical variables as number (n) and proportions. Student's *t*-test was used to compare two groups. Significance was set at P<0.05 (two-tailed significance test).

## Results

### Subjects' characteristics

According to criteria adopted by the World Health Organization (WHO) (9), 21 (49%) subjects had a BMI above normal (≥25 kg/m^2^): 15 (35%) were overweight (BMI of 25-29.9 kg/m^2^) and 6 (14%) were obese (BMI ≥30 kg/m^2^).

With regard to the overall self-reported alcohol consumption, 36 (84%) subjects did not use, or used an amount lower than 20 g daily. Four (9%) subjects had an intake between 20 and 40 g daily, while the other 3 (7%) had a daily intake over 40 g. Mild fibrosis predominated in the study sample (Table 1). The stage of liver fibrosis in one subject was unable to be assessed because of lack of tissue available for histopathological analysis. Notably, this subject presented with no clinical or laboratory evidence of cirrhosis.

**Table:**
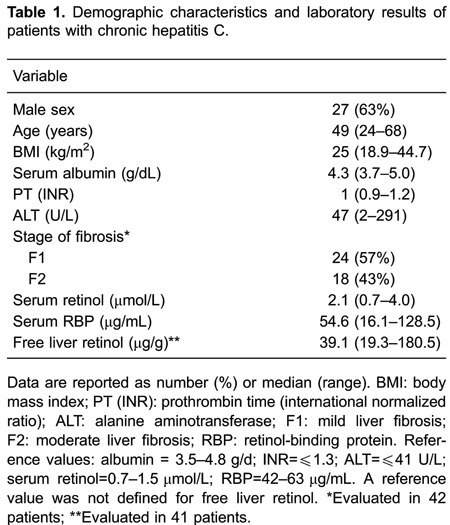

### Serum retinol, liver retinol, and RBP levels

According to criteria adopted by the WHO, vitamin A deficiency is defined as serum retinol levels ≤0.7 µmol/L (10). Thus, two subjects had a serum retinol level compatible with hypovitaminosis A. The RDR test was <20% in all of the patients, indicating adequate body vitamin A levels. The median weight of hepatic tissue fragments used for liver retinol quantification was 12 mg, ranging from 3 to 27 mg. Two fragments weighed less than 7 mg, which is considered the lower limit for reliable determination of hepatic vitamin A (11). Therefore, in these two cases, the hepatic tissue fragments were considered inadequate for liver retinol analysis. The median values of free liver retinol levels and the other vitamin A indicators are reported in Table 1.

### Serum retinol, liver retinol, and serum RBP levels according to BMI and the stage of liver fibrosis

To assess the effect of BMI on serum retinol, hepatic retinol, and RBP levels, subjects were divided into two groups: BMI <25 kg/m^2^ and BMI ≥25 kg/m^2^. Overweight or obese individuals had significantly higher serum retinol levels than those with a normal BMI (Table 2).

**Table:**
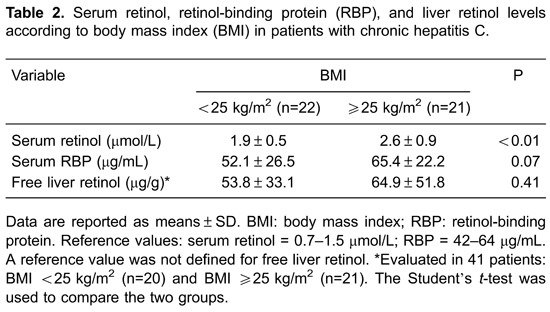

**Figure 1:**
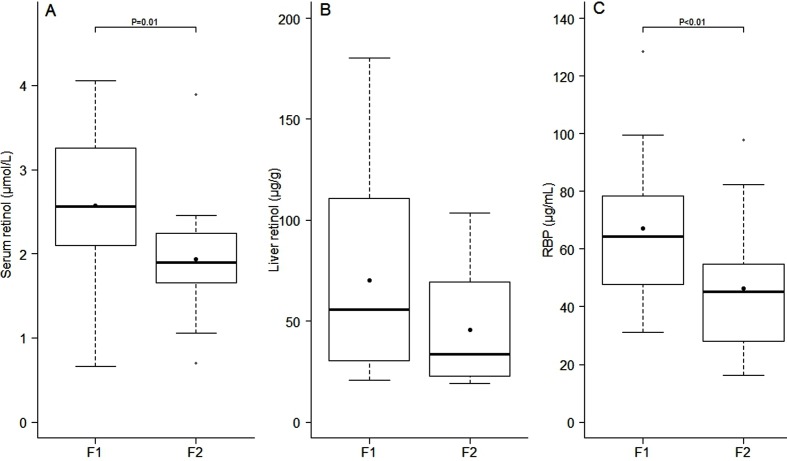
Serum retinol, liver retinol, and retinol-binding protein (RBP) levels according to the stage of liver fibrosis in the two groups according to the stage of liver fibrosis using the METAVIR score: mild liver fibrosis (F1) (n=24) and moderate liver fibrosis (F2) (n=18). *A*, Serum retinol levels were significantly lower in subjects with the F2 stage of fibrosis compared with those with the F1 stage of fibrosis (P=0.01, Student's *t*-test). *B*, No difference was detected in free liver retinol levels between the two groups (P=0.06, Student's *t*-test). *C*, Serum RBP levels were significantly lower in subjects with the F2 stage of fibrosis compared with those with the F1 stage of fibrosis (P<0.01, Student's *t*-test).

For analysis of the relationship between the stage of liver fibrosis and vitamin A indicators, patients were divided into groups of mild (F1) or moderate (F2). Serum retinol and RBP levels were significantly lower in subjects with moderate liver fibrosis than those with mild fibrosis (Figure 1). No difference in liver retinol levels was detected between these two groups.

## Discussion

Knowledge regarding vitamin A metabolism and liver disease is mainly based on studies that included patients with severe liver disease, and whose diverse etiologies were evaluated together (12-14). In the present study, only non-cirrhotic patients who were infected by HCV were evaluated. Because these patients were candidates for antiviral treatment, subjects with low alcohol intake were prevalent in the sample that was studied.

In the current study, 2 patients had serum retinol levels that were compatible with vitamin A deficiency. However, the RDR test was negative (<20%) in all of the subjects, indicating vitamin A sufficiency in the study sample. Serum retinol level tests have limitations in assessing an individual's vitamin A status because they are homeostatically controlled, and only reflect vitamin A liver stores at the extremes of deficiency or hypervitaminosis (4). The RDR test is considered as a more reliable test than a single serum retinol value. When comparing relative increases in serum retinol levels with the fasting level, after an oral dose of retinol palmitate, the RDR test reflects hepatic homeostatic mechanisms to maintain the supply of vitamin A to target tissues. When hepatic stores of vitamin A are depleted, after ingestion of an exogenous amount of retinol, the absorbed vitamin binds to the accumulated pool of RBP in the liver. This promptly releases the complex RBP-retinol (holo-RBP) in the blood circulation. In this situation, the difference between retinol values is over 20%. Thereafter, the RDR test indirectly reflects liver vitamin A stores (4). Peres et al. (14) assessed vitamin A stores in a group of Brazilian subjects with HCV-related chronic disease, and found inadequate vitamin stores according to the RDR test (RDR ≥20%) in 34% of the patients. Their sample differed from that of the present study in that the study subjects were mostly cirrhotic, where vitamin A deficiency is more prevalent.

In our study, significantly higher serum retinol levels were found in patients with a BMI ≥25 kg/m^2^ compared with those with a BMI <25 kg/m^2^. This finding may be due to differences in vitamin A intake (intake was not evaluated) between the two groups or related to adiposity. Aeberli et al. (15) found higher levels of serum retinol and RBP in overweight and obese children compared with those of normal-weight children. This finding is in line with the results from other authors who have described higher serum RBP levels in subjects with obesity (16,17), suggesting that adipose tissue plays a role in RBP secretion. Serum retinol is transported coupled to RBP (holo-RBP), which may explain the higher serum retinol concentrations detected in the overweight or obese patients. In the present study, although RBP levels were higher in subjects with a BMI ≥25 kg/m^2^than those with a BMI <25 kg/m^2^, this difference was not significant, which is possibly related to the small sample size.

In our study, serum RBP and retinol levels were significantly lower in patients with a fibrosis score of F2 compared with those with a score of F1. Gradual defects in the liver's ability to synthesize and/or secrete proteins as liver fibrosis progresses may explain this decrease in serum RBP levels. In agreement with our results, Huang et al. (18) found a significant decrease in RBP levels with the progression of stages of fibrosis in CHC patients. As previously mentioned, RBP acts as a blood carrier for retinol; therefore, a decrease in RBP may explain the lower serum retinol levels that were observed in patients with more advanced liver fibrosis. This result could also be explained by the loss of liver vitamin A stores in more advanced stages of fibrosis. During the fibrogenesis process, HSCs lose their contents of retinoids and differentiate from quiescent cells to myofibroblast-like cells (1). Retinol may also be consumed as a protective mechanism against oxidative stress, which has been shown in HCV-related chronic disease (19,20). The observed decrease in serum retinol and RBP levels with progression of hepatic fibrosis suggests a potential use of these markers in studies on noninvasive methods for assessment of liver fibrosis.

In the present study, free hepatic retinol levels were assessed in 41 patients, while two samples could not be analyzed because of the lack of liver tissue. Amédée-Manesme et al. (11) detected a correlation (r=0.96) of hepatic vitamin A concentrations in larger liver fragments that were obtained at autopsy with those in smaller fragments that were obtained by fine needle biopsy when fragments of up to 7 mg were used. In our sample, two hepatic tissue samples weighed below this limit. Therefore, they were excluded from the analysis of hepatic retinol because of the possibility of unreliable results. No difference was detected in free liver retinol levels in patients with moderate fibrosis compared with those with mild fibrosis. This finding may be related to the characteristics of the study sample, which consisted of subjects with less severe hepatic disease. Yadav et al. (20) found diminished liver retinol levels in CHC patients with moderate to severe fibrosis compared with those with mild fibrosis. Notably, because only free retinol was assessed in the present study, no inference can be made regarding total liver vitamin A stores. Ukleja et al. (12) found no difference in the values of liver retinol between cirrhotic patients and controls. However, liver retinyl esters and total vitamin A (free and esterified retinol) levels were significantly lower in cirrhotic patients than in controls in their study.

The lack of quantitation of liver retinol esters was a limitation of the present study. This impaired a more complete assessment of the effect of HCV-induced hepatic injury on total hepatic vitamin A stores. Another limitation to be considered is that, because assessment of oxidative stress and insulin resistance markers in these patients was not performed, we could not precisely estimate the effect of these factors on retinol and RBP concentrations.

However, we studied a group of subjects who are not often assessed using different and complementary tests, which provided a more reliable estimate of the body's vitamin A status. We conclude that subjects with HCV-related chronic disease in the milder stages of fibrosis and with low alcohol intake have vitamin A stores compatible with sufficiency. Therefore, this subgroup of subjects would not benefit from vitamin supplementation without a precise indication. However, progression of liver fibrosis is accompanied by a decrease in serum vitamin A.
